# Supplementary material for: A Fatal Case of Metastatic Pulmonary Calcification during the Puerperium
Source: Int J Mol Sci. 2022 Dec 1;23(23):15131. doi: 10.3390/ijms232315131 (PMC9735927; doi:10.3390/ijms232315131)
Supplement: Supplementary file 1 [file ijms-23-15131-s001.zip › ijms-2029906-supplementary.pdf]

Table S1a. *Blood test parameters - [normal laboratory values].*

**BNP** [<80 pg/ml]	**WBC** [4.30 – 10.50 x10^3]	**RBC** (4.20 – 5.80 x10^6)	**Hb** (12.0 - 17.50 g/dL)	**Hct** (37.0 – 52.0%)	**Plt** [130 – 400 x10^3]	**Neu** [40.0 – 74.0 %]	**Creatine** [0.6 – 1.2 mg/dL]	**Na$^+$** [136 – 146 meq/L]	**Ca^{++}** [2.5 – 4.5 mg/dL]	**P** [2.5 – 4.5 mg/dL]
132	6.31	4.21	12.5	31.5	257	84.8	5.42	128	13.5	5.0
**LDH** [0 – 248 U/L]	**PT INR** [0.8 – 1.20]	**APT** [17 – 35 sec.]	**APT ratio** [0.8 – 1.2]	**Fibr.** [150 – 450 mg/dL]	**D-Dim** (< 55 µg/L)	**PCR** [< 0.35 mg/dL]	**PCT** [<0.5 ng/mL]	**Azoto** [5 – 25 mg/dL]	**Prot.** [6.6 – 8.3 g/dL]	**Alb.** [3.5 – 5.2 g/dL]
301	2.36	36	1.2	537	3900	9.1	75.00	100	4.6	2.1

Table S1b. *Arterial gas tests.*

**Emergency Department admission**											
pH	PO_2 (mmHg)	PCO_2 (mmHg)	Na^+ (mmol/L)	K^+ (mmol/L)	Ca^{++} (mmol/L)	Lac (mmol/L)	Hb (g/dL)	Hct (%)	HCO_3^- (mmol/L)	P/F	BE (mmol/L)
7.31	57.5	34.4	125.0	3.9	2.0	4.2	12.7	42.9	16.9	273.7	-8.37
**ICU admission**											
pH	PO_2 (mmHg)	PCO_2 (mmHg)	Na^+ (mmol/L)	K^+ (mmol/L)	Ca^{++} (mmol/L)	Lac (mmol/L)	Hb (g/dL)	Hct (%)	-	P/F	BE (mmol/L)
6.77	48.6	101.1	132.4	7.36	2.025	9.3	11.4	43.5	-	48.6	-
